# A Meta-Analysis of the Effects of Different Exercise Modes on Inflammatory Response in the Elderly

**DOI:** 10.3390/ijerph191610451

**Published:** 2022-08-22

**Authors:** Haotian Zhao, Zhijian He, Hezhang Yun, Ruifu Wang, Chang Liu

**Affiliations:** 1 Department of Physical Education, Jiangnan University, Wuxi 214122, China; 2 Department of Sports Teaching and Research, Lanzhou University, Lanzhou 730000, China; 3 School of Sport Science, Beijing Sport University, Beijing 100084, China; 4 Department of Physical Education, Beijing Forestry University, Beijing 100083, China

**Keywords:** exercise modes, the elderly, inflammation, meta-analysis

## Abstract

The aim of this study was to investigate the effects of different exercise modes on improving inflammatory response in the elderly. For the research methodology, databases such as CNKI (China National Knowledge Infrastructure), Wanfang Data, Pubmed, Web of Science, and EBSCO were selected for searching. The Cochrane Risk of Bias (ROB) tool was used to evaluate the methodological quality of the included studies, and RevMan5.4.1 analysis software was applied for the statistical analysis. A total of 31 studies (20 randomized controlled trials and 11 self-controlled trials) with 1528 subjects were included. The results of this meta-analysis showed that aerobic exercise, resistance exercise, aerobic + resistance exercise, and HIIT all significantly reduced the levels of IL-6, TNF-α, and CRP in the elderly, and the improvement effects of aerobic + resistance exercise on IL-6, HIIT on TNF-α, and resistance exercise on CRP in the elderly were better than those of the other three exercise modes, respectively. In conclusion, aerobic exercise, resistance exercise, aerobic + resistance exercise, and HIIT all contribute to ameliorating the inflammatory status of the elderly, among which resistance exercise is a noteworthy exercise mode for the elderly to improve inflammatory status.

## 1. Introduction

As elderly individuals age, the increase in inflammatory factors often leads to a systemic and chronic inflammatory response state [1], which ultimately leads to poor vaccine efficacy, increased incidence of opportunistic infections, and a rising rate of morbidity and mortality in the elderly [2]. Regular participation in physical activity can help to decrease the incidence of different types of chronic diseases. It has been reported that the protective effect of exercise is associated with the improvement of immune function, which may regulate the status and function of macrophage polarization and contribute to the promotion of wound healing and sleep quality. Furthermore, regular exercise has been shown to be associated with a decreased risk of cancer and delayed tumorigenesis in the elderly [3,4,5].

Immune senescence is a progressive degeneration of the immune system function associated with human aging, which has been described as an increase in circulating concentrations of classical pro-inflammatory cytokines. Regarding systemic inflammation, elevated levels of the acute-phase protein C-reactive protein (CRP) and cytokines such as tumor necrosis factor alpha (TNF-α) and interleukin 6 (IL-6) have been found in the elderly [6]. These inflammatory markers not only accelerate the aging process but are also associated with an increased risk of developing cardiovascular diseases and cancers [7]. Currently, some studies have found that exercise reduces the levels of IL-6, TNF-α, and CRP, while others have reported that exercise does not improve the inflammatory status of the body [7]. Previous meta-analyses of the inflammatory response to exercise in the elderly included insufficient studies and the methodological quality of the included studies was relatively low [8,9,10]. In addition, that only one form of exercise was discussed, the inclusion of non-exercise forms of interventions in the study, and the wide age range of subjects and inaccurate results illustrate the limitations of the previous meta-analyses [8,11].

Although exercise has been shown to be effective in lowering IL-6, TNF-α, and CRP levels, there is no consensus on the improvement effects of different exercise modes on IL-6, TNF-α, and CRP in the elderly [8]. The different exercise modes studied here included aerobic exercise, resistance exercise, aerobic + resistance exercise, and HIIT. Aerobic exercise is a series of exercises that rely primarily on aerobic energy-production processes. “Aerobic” is defined as “involving, relating to, or requiring oxygen” and refers to the adequate use of oxygen to meet energy requirements through aerobic metabolism during exercise. Aerobic exercise is performed by repeating a series of light- to moderate-intensity activities over a long period of time. Resistance exercise involves physical exercise designed to improve strength and endurance. It is usually associated with weightlifting, and it can also include various training techniques such as calisthenic, isometric, and plyometric techniques. High-Intensity Interval Training (HIIT) is a training regimen that alternates short periods of high-intensity or explosive anaerobic exercise with short recovery periods until depleted, thus relying almost maximally on the anaerobic energy release system [12]. The purpose of this meta-analysis was to compare the effects of different exercise modes on the inflammatory response level in the elderly, so as to provide a reliable theoretical reference for the rational selection of exercise modes for the related diseases caused by systemic chronic inflammatory response in the elderly.

## 2. Materials and Methods

### 2.1. Selection and Exclusion Criteria of Literature

Inclusion criteria: (1) study subjects: physically and mentally healthy middle-aged and elderly people, free of diabetes, cardiovascular disease, and cancer, aged from 51 to 91 years old; (2) study types: randomized controlled trials or self-controlled trials; (3) intervention: exercise intervention; (4) outcome indicators: include one or more of IL-6, TNF-α, and CRP.

### 2.2. Searching Strategy

The literature search was conducted in accordance with the PRISMA (Preferred Reporting Items for Systematic Reviews and Meta-Analyses) guidelines. The title, abstract, keywords, and search fields were searched in CNKI (China National Knowledge Infrastructure), Wanfang Data, PubMed, Web of Science, and EBSCO, and the search period was until May 2022. The search was performed using Boolean operators combined with the following terms: “elderly”, “older”, “inflammatory reaction”, “inflammatory response”, “inflammatory factors”, “inflammatory”, “aerobic exercise”, “resistance exercise”, “high-intensity interval exercise (HIIT)”, “HIIT”, and “exercise intervention”. Additional searches were conducted within the reference lists of the included records.

### 2.3. Data Extraction and Quality Evaluation

#### 2.3.1. Data Extraction and Processing

The Endnote citation manager software was applied to conduct the literature screening. The first author of the literature, the year of publication, the sample-size number, the characteristics of the participants (gender, the number of male and female participants, age), the characteristics of the intervention (exercise modes, intervention protocol, duration, frequency), and the outcomes were extracted from the literature. Literature screening and data extraction were conducted independently by two researchers, who consulted each other after completion. If there was any disagreement, the studies would be submitted to a third researcher for further discussion and decision.

#### 2.3.2. Literature Quality Evaluation

Two researchers independently conducted the evaluation of the methodological quality and the risk of bias of the included studies according to the Cochrane Handbook 5.1.0, and the studies were assessed by the following aspects: (1) random sequence generation; (2) allocation concealment; (3) selective reporting; (4) blinding of participants and personnel; (5) blinding of outcome assessment; (6) incomplete outcome data; and (7) other biases. Based on the evaluation criteria, the studies were classified as “low risk”, “medium risk”, “high risk” or “unknown risk”. For the self-controlled trials, the ROBINS-I 2.0 (Risk of Bias in Non-randomized Studies of Interventions) was used to evaluate the methodological quality and the risk of bias.

## 3. Results

### 3.1. General Results of Selected Research Literature

**Figure 1:**
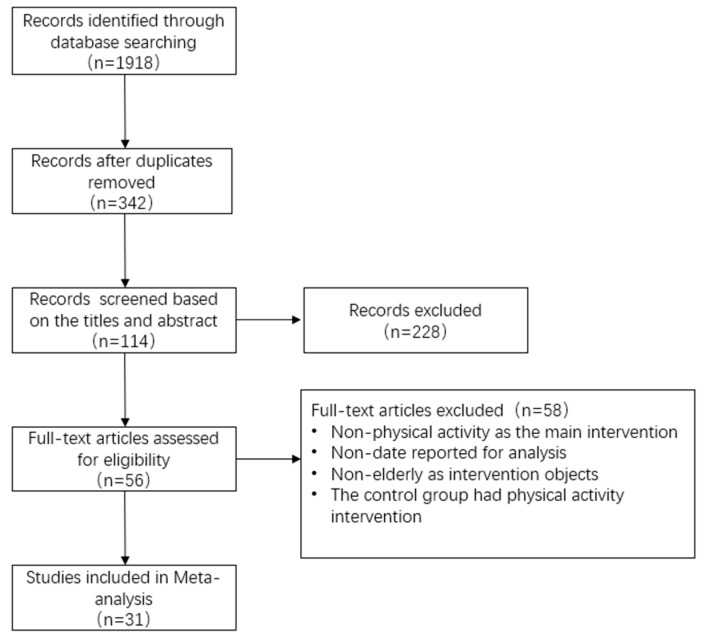
Literature selection process.

After screening the literature strictly according to the established uniform inclusion and exclusion criteria, we found that the sample size of the randomized controlled trials was limited and that many of the screened studies were self-controlled trials. Considering that the limited number of randomized controlled trials may reduce the reliability of the meta-analysis, the low-risk and high-quality self-controlled trials and studies with participants aged 51 years or older were also included in the study. For the self-controlled trials, meta-analyses were performed to compare the differences between “pre-exercise intervention” and “post-exercise intervention” data. After the final screening, 31 articles with a total sample size of 1528 were included, and the studies were mainly conducted in healthy elderly people without diabetes, cardiovascular disease, and cancer. The flow chart of the literature screening is shown in Figure 1.

### 3.2. General Features of the Selected Research Literature

#### 3.2.1. General Information of Each Study

Through rigorous screening according to the inclusion and exclusion criteria, 20 randomized controlled trials (RCT) and 11 self-controlled trials (SCT) ultimately met the inclusion criteria for the meta-analysis, and a total of 1528 subjects were included in these 31 trials. Among the included studies, 11 studies included aerobic exercise (AE), 16 studies included resistance exercise (RE), 3 studies included aerobic + resistance exercise (AE+RE), and 2 studies included HIIT (see Table 1).

#### 3.2.2. Quality Evaluation of the Selected Literature

**Figure 2:**
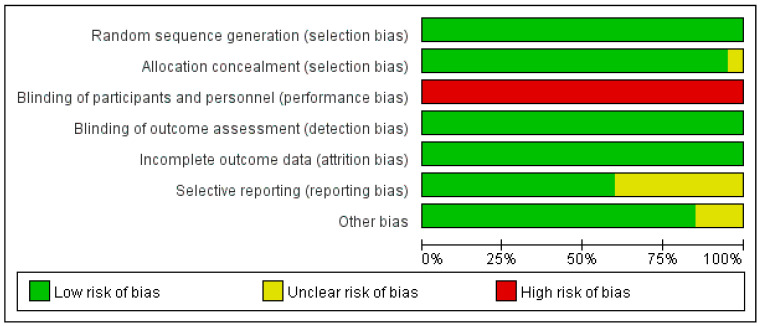
Risk of bias graph.

**Figure 3:**
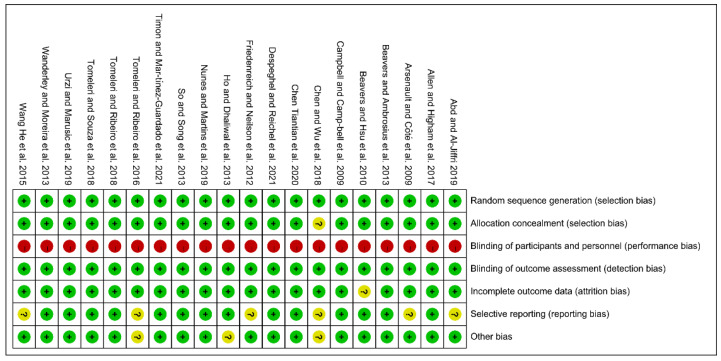
Risk of bias summary [14,15,16,17,19,20,21,22,25,30,32,33,34,35,36,37,38,40,41].

A total of 31 studies, including 20 randomized controlled trials and 11 self-controlled trials, were included in the study. Due to the characteristics of the exercise intervention, the assessment of performance bias (blinding of participants and personnel) was excluded from the evaluation. Finally, the risk of bias in all of the included 20 RCT studies was moderate, with 1 study having a moderate risk of selection bias, 8 studies having a moderate risk of reporting bias, and 3 studies having a moderate risk of other bias. After the exclusion of the bias due to deviations from the intended interventions, all 11 self-controlled trials had a low-to-moderate risk of bias. (Figure 2 and Figure 3 and Table 2).

### 3.3. Effects of Different Exercise Modes on IL-6 in the Elderly

**Figure 4:**
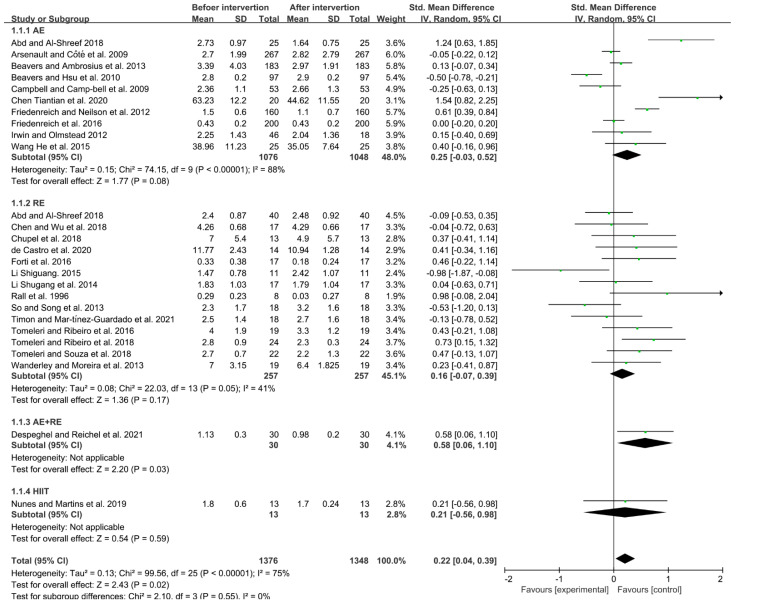
Effects of different exercise modes on IL-6 in the elderly [7,13,14,15,16,17,18,19,21,22,24,25,26,27,28,31,32,33,34,36,38,39,40,41].

Due to the high heterogeneity between the studies (I^2^ = 75%, *p* < 0.05), a random-effects model was used for analysis. The results indicated that exercise could significantly reduce the level of IL-6 in the elderly (SMD = 0.22, 95% CI = 0.04~0.39, *p* < 0.05). Subgroup analysis showed that aerobic exercise, resistance exercise, aerobic + resistance exercise, and HIIT could all reduce the level of IL-6 in the elderly (aerobic exercise: SMD = 0.25, 95% CI = −0.03~0.52, *p* = 0.08; resistance exercise: SMD = 0.16, 95% CI = −0.07~0.39, *p* = 0.17). Meanwhile, both aerobic + resistance exercise and HIIT could lower the level of IL-6 in the elderly (aerobic + resistance exercise: SMD = 0.58, 95% CI = 0.06~1.10, *p* < 0.05; HIIT: SMD = 0.21, 95% CI = −0.56~0.98, *p* = 0.59). The aerobic + resistance exercise mode had a better effect than the other exercise modes in reducing IL-6 in the elderly (Figure 4).

### 3.4. Effects of Different Exercise Modes on TNF-α in the Elderly

**Figure 5:**
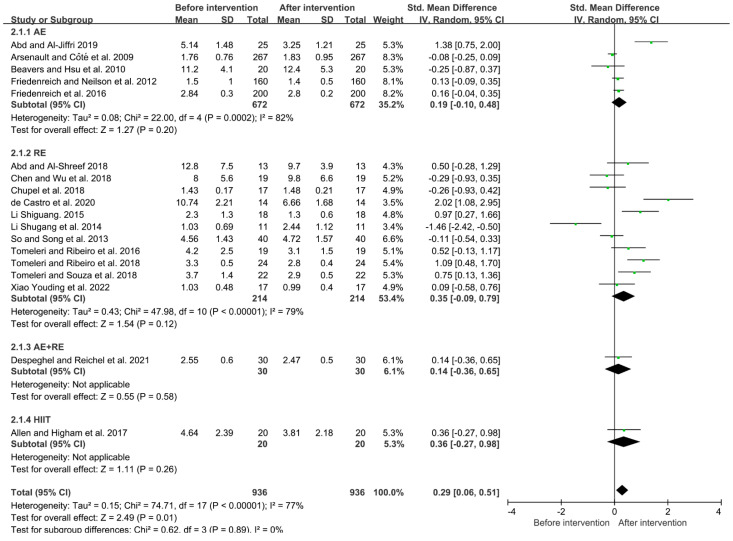
Effects of different exercise modes on TNF-α in the elderly [7,14,16,17,21,23,24,26,28,30,31,32,33,34,35,39,40,42].

Due to the high heterogeneity among the studies (I^2^ = 77%, *p* < 0.05), the random-effects model was used for analysis. The results showed that exercise could significantly reduce the level of TNF-α in the elderly (SMD = 0.29, 95% CI = 0.06~0.51, *p* < 0.05). Subgroup analysis showed that aerobic exercise, resistance exercise, aerobic + resistance exercise, and HIIT could all reduce the level of TNF-α in the elderly (aerobic exercise: SMD = 0.19, 95% CI = −0.10~0.48, *p* = 0.20; resistance exercise: SMD = 0.35, 95% CI = −0.09~0.79, *p* = 0.12; aerobic + resistance exercise: SMD = 0.14, 95% CI = −0.36~0.65, *p* = 0.58; HIIT: SMD = 0.36, 95% CI = −0.27~0.98, *p* = 0.26). The HIIT mode had a better effect on the reduction in TNF-α in the elderly compared to other exercise modes (Figure 5).

### 3.5. Effects of Different Exercise Modes on CRP in the Elderly

**Figure 6:**
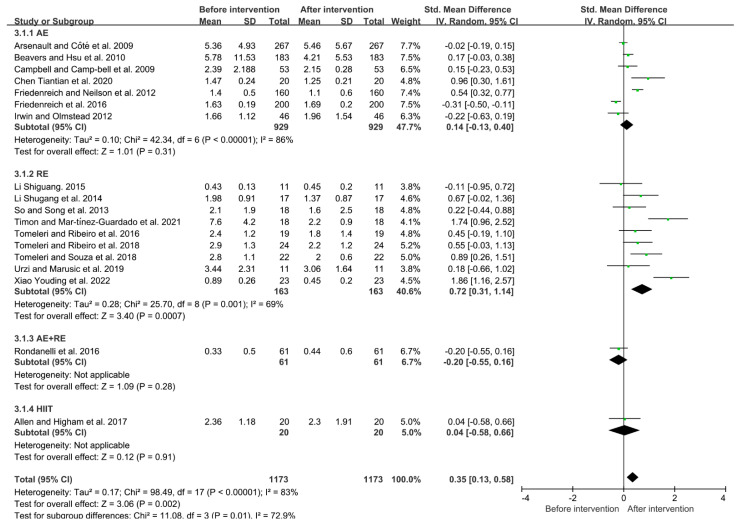
Effects of different exercise modes on CRP in the elderly [14,15,16,17,18,21,23,24,26,28,29,30,33,34,37,38,41,42].

Due to the high heterogeneity between the studies (I^2^ = 83%, *p* < 0.01), the random-effects model was used for analysis. The results showed that exercise could significantly reduce the level of CRP in the elderly (SMD = 0.26, 95% CI = 0.08~0.43, *p* < 0.05). Subgroup analysis showed that aerobic exercise, resistance exercise and HIIT could all reduce the level of CRP in the elderly (aerobic exercise: SMD = 0.14, 95% CI = −0.13~0.40, *p* = 0.31; resistance exercise: SMD = 0.72, 95% CI = 0.31~1.14, *p* < 0.01; aerobic + resistance exercise: SMD = −0.20, 95% CI = −0.55~0.16, *p* = 0.28; HIIT: SMD = 0.04, 95% CI = −0.58~0.66, *p* = 0.91). The resistance exercise mode had a better effect than the other exercise modes in decreasing CRP in the elderly (Figure 6).

## 4. Discussion

The results showed that both aerobic exercise and resistance exercise reduced the levels of IL-6, TNF-α, and CRP in the elderly and that resistance exercise had a better effect than aerobic exercise, which is consistent with previous studies [8]. These results may be due to the fact that resistance exercise can increase the antioxidant capacity of skeletal muscle in the elderly [9] through up-regulating superoxide dismutase (SOD), catalase (CAT) and glutathione peroxidase (GPX), thus improving the anti-inflammatory capacity [43].

Aerobic exercise, resistance exercise, and aerobic + resistance exercise all have a better effect than HIIT in reducing the level of IL-6 in the elderly, which is similar to previous studies [44]. It is speculated that HIIT may lead to an increase in the expression level of the pro-inflammatory factor IL-6 (Table 3). However, it was found that the increase in IL-6 induced by HIIT had no adverse effects on health [45], as the increase in IL-6 induced by HIIT may have anti-inflammatory properties. This speculation was confirmed in the subsequent discussion on TNF-α.

As for TNF-α, we found that different exercise modes could all reduce the level of TNF-α in the elderly, of which HIIT had the most significant effect (Table 2). In addition to the increase in anti-inflammatory factor IL-6 induced by HIIT, there is also a prolonged excessive oxygen consumption following the end of HIIT [46]. In the state of excessive oxygen consumption, the rate of lipid metabolism increases. Given that adipose tissue continuously secretes inflammatory factors, we speculate that HIIT can reduce the level of TNF-α by improving lipid metabolism in the elderly. In addition, we observed that aerobic exercise did not have a significant effect on ameliorating the level of TNF-α; this may be because of the short intervention period (6 months to 12 months) included in the studies. Some studies suggested that aerobic exercise takes at least one year to result in a significant reduction in the level of TNF-α [47].

Finally, the results showed that the improvement effect of resistance exercise on CRP is more significant than in other exercise modes (Table 2). Unlike IL-6 and TNF-α, CRP is an acute-phase plasma protein not only synthesized by the liver but also by adipocytes. One of its functions is to send signals to the immune system, instructing which cells participate in the defense response to infections and the inflammatory response of the body [48]. Like other cytokines, CRP is associated with cardiovascular disease and diabetes and is one of the factors of chronic inflammatory response in the human body. Resistance exercise can stimulate skeletal muscle metabolism by activating the 5′ adenosine monophosphate-activated protein kinase (AMPK) system [9]. Through this, it can help regulate hepatic glycogen synthesis and fat decomposition, delay muscle reduction, and increase body metabolism throughout the day, thus reducing CPR levels and ameliorating inflammation levels.

## 5. Limitations of Current Research

Despite our strict adherence to the procedures set out in PRISMA, the literature related to AE + RE and HIIT interventions is limited for our study, resulting in the possibility of heterogeneity in the results.

The elderly population itself may have elevated levels of inflammatory factors due to aging, which may have interfered with the assessment of inflammatory factor changes; the exercise interventions involved multiple movements and forms, and the training environment was not fixed, which may have biased the results due to other factors.

## 6. Conclusions

Based on the results of this meta-analysis, we can draw several conclusions. Aerobic exercise, resistance exercise, aerobic + resistance exercise, and HIIT can all contribute to ameliorating the level of inflammation in the elderly and reduce the levels of pro-inflammatory factors IL-6, TNF-α and CRP.

In particular, aerobic exercise, resistance exercise, and aerobic + resistance exercise all have a better effect than HIIT on reducing the level of IL-6 in the elderly, while HIIT is superior to other exercise methods in improving the anti-inflammatory characteristics of the body. Meanwhile, resistance exercise was superior to other exercise modalities in improving the inflammatory response, which may be a noteworthy exercise modality for improving inflammatory status in the elderly.

## Figures and Tables

**Table 1:** General features of selected research literature.

First Author	Year	Type of Study, Health Condition	Age (Years)		Sample Size (n)		Intervention	Frequency	Duration	Outcome
			Control Group	Intervention Group	Control Group	Intervention Group				
Rall and Roubenoff et al. [13]	1996	SCT, Healthy		70.3 ± 5.0		8	RE	1/2W	12 W	IL-6
Arsenault and Côté et al. [14]	2009	RCT, Overweight/Obesity	57.2 ± 6.1	57.3 ± 6.6	82	267	AE	3–4/W	6 M	IL-6, TNF-α, CRP
Campbell and Campbell et al. [15]	2009	RCT, Overweight	60.9 ± 6.8	60.5 ± 7.0	62	53	AE		12 M	CRP, IL-6
Beavers and Hsu et al. [16]	2010	RCT, Healthy	77.0 ± 4.4	76.4 ± 4.1	186	183	AE	3/W	12 M	IL-6, CRP
Friedenreich and Neilson et al. [17]	2012	RCT, Healthy	60.6 ± 5.7	61.2 ± 5.4	160	160	AE	5/W	12 M	IL-6, TNF-α, CRP
Irwin and Olmstead [18]	2012	SCT, Healthy		70.7 ± 5.9		46	AE	2/W	25 W	IL-6, CRP
Beavers and Ambrosius et al. [19]	2013	RCT, Overweight/Obesity	60–79	60–79	93	97	AE	5/W	18 M	IL-6
Ho and Dhaliwal et al. [20]	2013	RCT, Overweight/Obesity	52 (40–66)	53 (43–64)	16	17	AE+RE	3/W	12 M	IL-6, TNF-α
So and Song et al. [21]	2013	RCT, Healthy	68.4 ± 5.8	71.6 ± 5.5	22	18	RE	3/W	12 W	IL-6, TNF-α
Wanderley and Moreira et al. [22]	2013	RCT, Healthy	67.8 ± 5.5	67.3 ± 4.9	11	19	RE	2/W 8/M		IL-6, TNF-α
				69.9 ± 5.7		20	AE			
Tomeleri and Ribeiro et al. [23]	2016	RCT, Healthy	69.5 ± 4.7	66.8 ± 3.2	19	19	RE	3/W	8 W	IL-6, TNF-α, CRP
Li Shugang et al. [24]	2014	SCT, Healthy		65.65 ± 3.14		17	RE	2/W	16 W	IL-6, TNF, CRP
Wang He et al. [25]	2015	RCT, Healthy	63.39 ± 3.43	63.53 ± 3.12	20	25	AE	5/W	6 M	IL-6, TNF
Li Shiguang er al. [26]	2015	SCT, Healthy		70.63 ± 3.93		11	RE	3/W	12 W	IL-6, TNF, CRP
Forti and Van Roie et al. [27]	2016	SCT, Healthy		67.86 ± 4.36		17	RE	3/W	12 W	IL-6
Friedenreich and O’Reilly et al. [28]	2016	SCT, Healthy		59.4 ± 4.8		200	AE	5/W	12 M	IL-6, TNF-α, CRP
Rondanelli and Klersy et al. [29]	2016	SCT, Healthy		80.21 ± 8.54		61	AE + RE	5/W	12 W	CRP
Allen and Higham et al. [30]	2017	RCT, Healthy	49.2 ± 6.1		14	20	HIIT	3/W	9 W	TNF-α, CRP
Chupel and Minuzzi et al. [31]	2018	SCT, Healthy		83.5 ± 7.3		13	RE	2/W	14 W	IL-6, TNF-α
Chen and Wu et al. [32]	2018	RCT, Healthy	68.3 ± 2.8	66.7 ± 5.3	16	17	RE	2/W	8 W	CRP, IL-6, TNF-α
Tomeleri and Ribeiro et al. [33]	2018	RCT, Healthy	68.8 ± 4.6	71.0 ± 5.4	22	24	RE	3/W	12 W	IL-6, TNF-α, CRP
Tomeleri and Souza et al. [34]	2018	RCT, Healthy	68.8 ± 4.9	72.1 ± 6.3	23	22	RE	3/W	18 W	IL-6, TNF-α, CRP
Abd and Al-Shreef [7]	2018	SCT, Healthy		65.96 ± 3.42		40	RE	3/W	6 M	IL-6, TNF-α
Abd and Al-Jiffri [35]	2019	RCT, Healthy		61–67	25	25	AE	3/W	6 M	IL-6, TNF-α
Nunes and Martins et al. [36]	2019	RCT, Healthy	62.9 ± 2.25	62.3 ± 2.08	13	13	HIIT	3/W	12 W	IL-6
Urzi and Marusic et al. [37]	2019	RCT, Healthy	88.9 ± 5.3	84.4 ± 7.7	9	11	RE	3/W	12 W	CRP
Chen Tiantian et al. [38]	2020	RCT, Healthy	63.59 ± 2.21	64.75 ± 2.89	10	20	AE	6/W	12 M	IL-6, CRP
de Castro and Da et al. [39]	2020	SCT, Healthy		67.36 ± 7.13		14	RE		Dis	IL-6, TNF-α
Despeghel and Reichel et al. [40]	2021	RCT, Healthy	69.8 ± 4.4	70.4 ± 5.3	10	30	AE + RE	-	6 W	IL-6, TNF-α
Timon and Martínez-Guardado et al. [41]	2021	RCT, Healthy	70.5 ± 4.0	70.3 ± 3.3	19	18	RE	3/W	24 W	IL-6, CRP
Xiao Youding et al. [42]	2022	SCT, Healthy		70.79 ± 4.91		23	RE	3/W	16 W	TNF-α, CRP

W: week(s); M: month(s); Dis: disposable.

**Table 2:** Results of quality evaluation of included self-controlled trial literature.

Study	Bias Due to Confounding	Bias in Selection of Participants into the Study	Bias in Classification of Interventions	Bias Due to Deviations from Intended Interventions	Bias Due to Missing Data	Bias in Measurement of Outcomes	Bias in Selection of the Reported Result
Abd and Al-Shreef [7]	Low risk	Low risk	Low risk	High risk	Low risk	Low risk	Low risk
Chupel and Minuzzi et al. [31]	Low risk	Low risk	Low risk	High risk	Low risk	Low risk	Low risk
de Castro and Da et al. [39]	Low risk	Low risk	Low risk	High risk	Low risk	Low risk	Low risk
Forti and Van Roie et al. [27]	Low risk	Low risk	Low risk	High risk	Low risk	Low risk	Low risk
Friedenreich and O’Reilly et al. [28]	Low risk	Low risk	Low risk	High risk	Low risk	Low risk	Low risk
Irwin and Olmstead [18]	Low risk	Low risk	Low risk	High risk	Low risk	Low risk	Low risk
Rall and Roubenoff et al. [13]	Medium risk	Low risk	Low risk	High risk	Low risk	Low risk	Low risk
Rondanelli and Klersy et al. [29]	Low risk	Low risk	Low risk	High risk	Low risk	Low risk	Low risk
Li Shiguang et al. [26]	Low risk	Low risk	Low risk	High risk	Low risk	Medium risk	Low risk
Li Shugang et al. [24]	Low risk	Low risk	Low risk	High risk	Low risk	Medium risk	Low risk
Xiao Youding et al. [42]	Medium risk	Low risk	Low risk	High risk	Low risk	Medium risk	Low risk

**Table 3:** Subgroup analysis of the effect of different modes on inflammatory response in the elderly.

Subgroup	IL-6				TNF-α				CRP			
Intervention	Effect Size	95% CI	n	I^2^	Effect Size	95% CI	n	I^2^	Effect Size	95% CI	n	I^2^
AE	0.25	−0.03, 0.52	1076	88%	0.19	−0.10, 0.48	672	82%	0.14	−0.13, 0.40	929	86%
RE	0.16	−0.07, 0.39	257	41%	0.35	−0.09, 0.79	214	79%	0.72	0.31, 1.14	163	89%
AE + RE	0.58	0.06, 1.10	30	—	0.14	−0.36, 0.65	30	—	−0.2	−0.55, 0.16	61	—
HIIT	0.21	−0.56, 0.98	13	—	0.36	−0.27, 0.98	20	—	0.04	−0.58, 0.66	20	—
Total	0.22	0.04, 0.39	1376	75%	0.29	0.06, 0.51	936	77%	0.35	0.13, 0.58	1173	83%

## Data Availability

Not applicable.
